# Quantitative proteome of midgut, Malpighian tubules, ovaries and fat body from sugar-fed adult *An. stephensi* mosquitoes

**DOI:** 10.1016/j.dib.2018.08.189

**Published:** 2018-09-05

**Authors:** Sreelakshmi K. Sreenivasamurthy, Gourav Dey, Manish Kumar, Ajeet Kumar Mohanty, Ashwani Kumar, T.S. Keshava Prasad

**Affiliations:** a Institute of Bioinformatics, International Tech Park, Bangalore 560066, India; b Manipal Academy of Higher Education, Manipal 576104, India; c Center for Systems Biology and Molecular Medicine, Yenepoya Research Centre, Yenepoya (Deemed to be University), Mangalore 575018, India; d ICMR-National Institute of Malaria Research, Field Station, Campal, Panaji, Goa 403001, India

## Abstract

The data presented in this article is associated with the quantitative proteomic analysis of four mosquito tissues – midgut, Malpighian tubules, ovaries and fat body from female *Anopheles stephensi* mosquitoes. To identify the proteins that were expressed in a tissue-specific manner, the four mosquito tissues were labelled with iTRAQ labels and analyzed using a high-resolution mass spectrometer. Database searches of the 1,10,616 raw spectra from 23 peptide fractions resulted in the identification of 84,733 peptide spectrum matches corresponding to 16,278 peptides and 3372 proteins. Of these, 959 proteins were found to be differentially expressed across the tissues. Gene ontology-based bioinformatic analysis of the differentially expressed proteins are also provided in the article. The data in this article has been deposited in the (ProteomeXchange) Consortium via the PRIDE repository and can be accessed through the accession ID, PXD001128.

**Specifications table**

**Table:** 

Subject area	Biology
More specific subject area	Vector Biology, proteomics
Type of data	High-resolution mass spectrometry based quantitative proteomics data in the form of excel files and figures
How data was acquired	The data was acquired on LTQ-Orbitrap Velos mass spectrometer from Thermo Scientific, Bremen, Germany. The data was searched using the Proteome Discoverer 2.1 employing Sequest and Mascot search engines (Matrix Science, London, UK; version 2.2).
Data format	Analyzed data in the form of excel files
Experimental factors	Different tissues from adult female *An. stephensi* mosquitoes
Experimental features	Dataset from iTRAQ labelled peptides extracted from midgut, Malpighian tubules, ovaries and fat body tissues dissected from sugar-fed female *An. stephensi* mosquitoes
Data source location	Bangalore and Goa, India
Data accessibility	The raw data and the searched files in the form of msf is accessible from the ProteomeXchange Consortium
	(http://proteomecentral.proteomexchange.org) via the PRIDE partner repository with the dataset identifier PXD001128.
	The data from the analysis is also provided in the form of excel tables through this article.

**Value of the data**

• This dataset provides the quantitative proteomic profile of four female *An. stephensi* mosquito tissues including midgut, Malpighian tubules, ovaries and fat body
• The dataset is a major resource to identify the tissue-restricted proteins expressed in these mosquito tissues
• The peptides identified in the dataset is proof of translation for the computationally predicted coding regions in the *An. stephensi* genome
• The dataset has aided in the identification of novel *An. stephensi* proteins and protein coding regions of the genome

## 1 Data

**Fig. 1:**
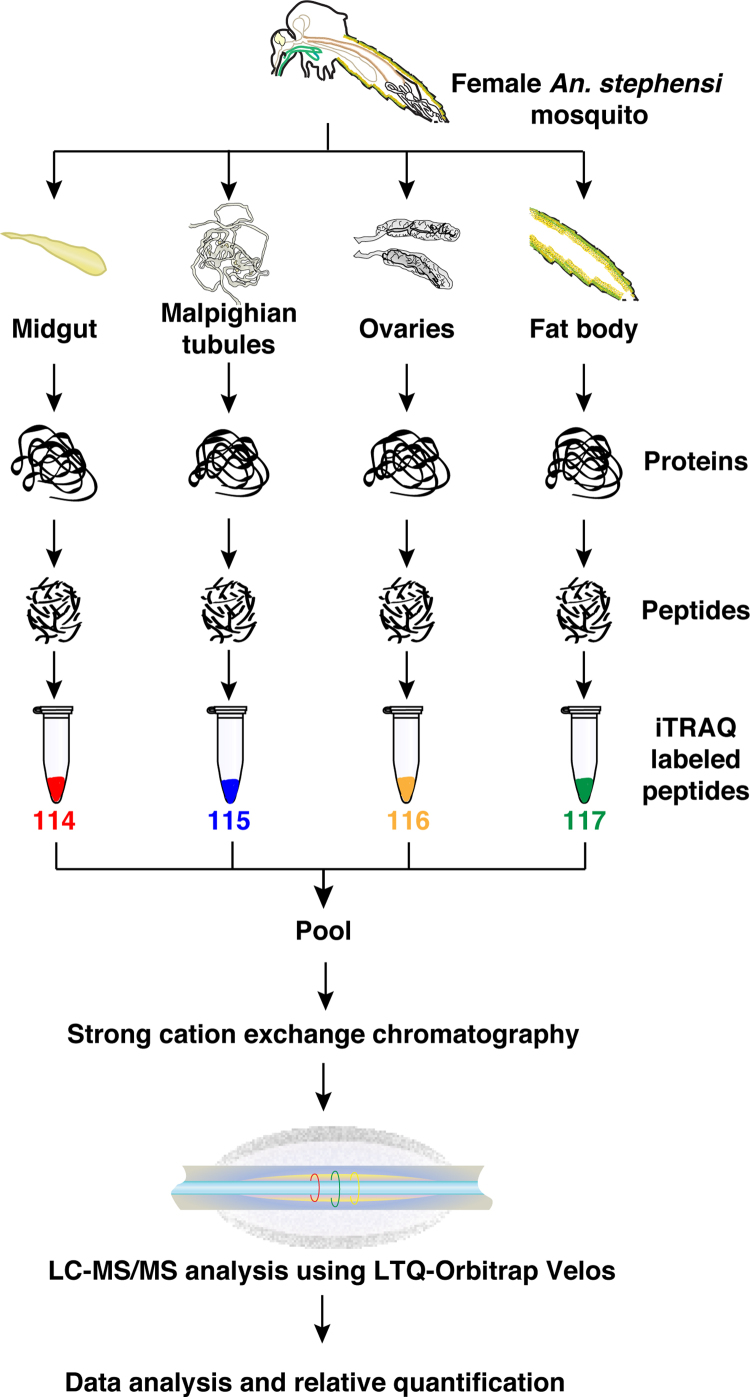
Workflow of the iTRAQ-based proteomic analysis of four tissues – midgut, Malpighian tubules, ovaries and fat body.

**Fig. 2:**
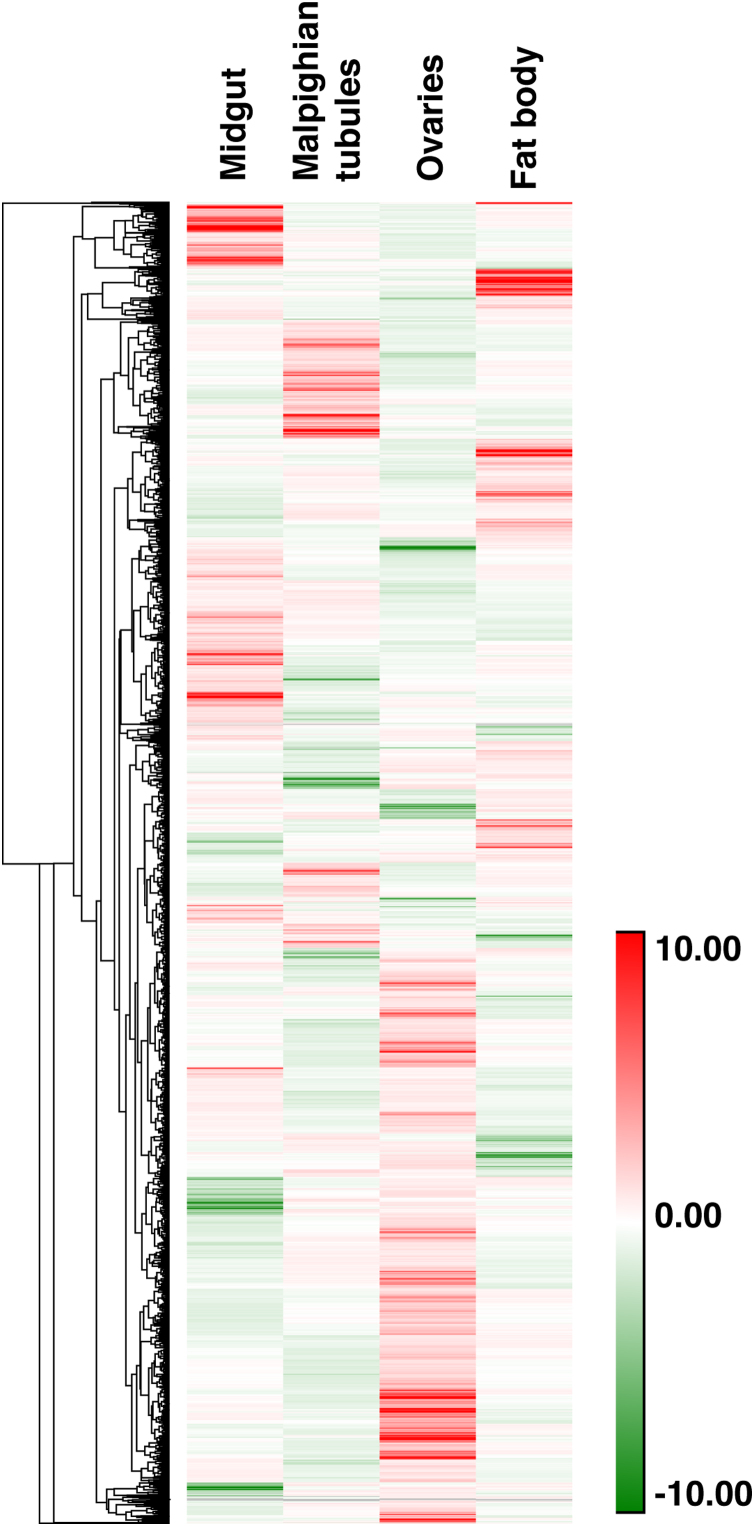
Heatmap representation of the expression of proteins in the four (midgut, Malpighian tubules, ovaries and fat body) *An. stephensi* tissues.

This article encompasses the proteomic data from an iTRAQ-based quantitative proteomic analysis of four female adult *Anopheles stephensi* mosquito tissues namely, midgut, Malpighian tubules, ovaries and fat body. The raw data, along with the search files have been deposited in the web-based online repository PRIDE from the ProteomeXchange consortium and is also part of our previously published article entitled - “Integrating transcriptomics and proteomics data for accurate assembly and annotation of genomes” [1]. The data was obtained from the high-resolution mass spectrometry-based analysis of 23 peptide fractions from a strong cation exchange (SCX) chromatography-based high-performance liquid chromatography fractionation method. To identify the differentially expressed proteins in these tissues, proteins isolated from tissues dissected from adult female *An. stephensi* mosquitoes were digested with trypsin and the resulting peptides were labelled with iTRAQ labels before fractionating and analyzing on the mass spectrometer as depicted in the Fig. 1. The list of protein and peptide identifications from the combined searches of the 23 fractions using Sequest and Mascot search engines are provided in the Supplementary Table 1. A summary of the proteins identified to be differentially expressed in each of these tissue with respect to the others is provided in Table 1, while the list of proteins along with their Gene Ontology information is provided as Supplementary Table 2. Gene Ontology analysis of the differentially expressed proteins revealed proteins involved in different biological processes and molecular functions. Fig. 2 provides a heatmap representation of the protein expression in the four *An. stephensi* tissues.

**Table 1:** Summary of the proteins identified to be differentially expressed across different tissues of female *An. stephensi* sugar-fed mosquitoes.

**Upregulated in →**	***Midgut***	***Malpighian tubules***	***Ovaries***	***Fat body***	
**Downregulated in ↓**					
**Midgut**	*vs.*	401	644	399	Midgut
**Malpighian tubules**	480	*vs.*	690	402	Malpighian tubules
**Ovaries**	509	426	*vs.*	404	Ovaries
**Fat body**	383	332	548	*vs.*	Fat body
	*Midgut*	*Malpighian tubules*	*Ovaries*	*Fat body*

## 2 Experimental design, materials and methods

### 2.1 Mosquito rearing and dissection

*An. stephensi* mosquitoes of the Type form strain were grown at the National Institute of Malaria Research laboratory field station in Goa. Female adult mosquitoes were maintained in cages at ambient temperatures of 27 °C ± 2 °C and about 70% humidity with 12-h light and dark periods. Two to four-day old female mosquitoes were captured and the four tissues (midgut, Malpighian tubules, ovaries and fat body) were dissected under a stereomicroscope and collected in four separate tubes. The individual tissues dissected from about 500 female mosquitoes were collected in a microcentrifuge kept on ice. The tissues were then washed thrice with sterile PBS and stored by freezing at − 80 °C until protein extraction.

### 2.2 Protein extraction and sample preparation

The frozen tissues were lysed by sonicating in 0.5% SDS buffer using a probe sonicator and centrifuged at 14,000 rpm for 20 min at 4 °C to separate the cell debris from the lysate. The supernatant containing the proteins was collected after centrifugation and the amount of protein was estimated using the bicinchoninic acid (BCA) assay. Protein estimation was visually confirmed by loading equal amounts of proteins on an SDS-PAGE for normalization purposes. Equal amounts of proteins (about 80 µg) from each tissue was reduced using tris-(2-carboxyethyl) phosphine (TCEP) at 60 °C for 1 h and further alkylated using methylmethanethiosulfonate (MMTS) for 10 minutes at room temperature before digesting with sequencing grade trypsin (Promega) at 37 °C overnight. The resulting peptides were then vacuum dried and labeled with 4-plex iTRAQ labels, which generate reporter ions peaks of m/z 114, 115, 116 and 117 upon ionization in the mass spectrometer for midgut, Malpighian tubules, ovaries and fat body derived peptides, respectively. The iTRAQ labeled peptides were pooled and checked for the labeling efficiency before proceeding with fractionation using SCX chromatography.

### 2.3 Peptide fractionation and cleanup

Labeled peptides from each tissue was pooled in equal amounts and subjected to charge-based fractionation using the SCX liquid chromatography column packed with 5 µ, 200 Ǻ polysulfoethyl A matrix (PolyLC) on an Agilent 1200 infinity series HPLC system. An increasing salt gradient of up to 350 mM KCl was used over 50 minutes to fractionate the peptides. The salt content in the individual fractions were removed using the stage tips packed with the C18 matrix (3 M Empore high-performance extraction disks). The peptides bound to the C18 matrix were eluted using 80% acetonitrile, after washing away the unbound salts from the columns. The cleaned mass spectrometry-ready peptides were used for the mass spectrometry analysis.

### 2.4 Mass spectrometry and data analysis

Cleaned and dried peptide fractions were reconstituted in 0.1% formic acid solution and injected in to the mass spectrometry through the Easy-nanoLCII (Thermo Scientific), after partial enrichment of the peptides on a pre-column followed by further separation of peptides using an analytical column. The pre-column and the analytical column were packed in-house with the magic AQ C18 material (Michrom Bioresources) of 5 µ and 3 µ, to a length of about 2 cm and 10 cm, respectively. The eluting peptides were sprayed on to the mass spectrometer using a nano electro spray emitter tip of 10 µ (New Objective) at 2 kV and 220 °C. The data acquisition was performed in an Orbitrap in a data dependent manner by selecting 15 most intense precursor ions for MS2 level fragmentation from each MS scan. The resolution of the mass analyzer was set at 60,000 at 400 m/z and 15,000 for the acquisition of MS and MS/MS scans.

The raw data in the forms of mass spectra was searched against the theoretical spectra generated from the protein database of *An. stephensi* (ASTEI 2.2) released by VectorBase. The searches were carried out on the Proteome Discoverer suite using the Sequest and Mascot search engines to identify the peptides and proteins. The intensity of the reporter ion peaks was used for the quantification of relative abundance of a given peptide and thereby the corresponding proteins in a given tissue. The search parameters employed for the database searches included the methylthiol modification of cysteine, iTRAQ labels on the peptide N-terminus and lysine residues as static modifications and oxidation of methionine as variable modification. A mass tolerance of 20ppm and 0.05 Da was applied for the precursor and fragment ion matching with a signal to noise ratio of 1.5 for a precursor mass ranging between 350 and 10,000 Da. The minimum peptide length of 7 amino acids was used with a false discovery rate of 1% applied to the results at peptide level. Only the unique and unambiguous peptide identifications for a given protein were used for the quantification purposes. Gene ontology information of the proteins were obtained from VectorBase and used to determine the processes in which the differentially expressed proteins were involved in.

## Appendix A Supplementary material

Supplementary material **Supplementary File 1:** Compiled list of proteins identified from 23 fractions of the iTRAQ-based proteomic analysis along with their scaled abundances in each tissue.

Supplementary material Supplementary Table 2**:** Summary of the differentially expressed proteins in each tissue compared to the other tissues along with the biological processes they are involved in based on their Gene Ontology analysis.
